# Different Permeability of Potassium Salts across the Blood-Brain Barrier Follows the Hofmeister Series

**DOI:** 10.1371/journal.pone.0078553

**Published:** 2013-10-28

**Authors:** Gian Luca Breschi, Massimo Cametti, Alfonso Mastropietro, Laura Librizzi, Giuseppe Baselli, Giuseppe Resnati, Pierangelo Metrangolo, Marco de Curtis

**Affiliations:** 1 Unit of Epileptology and Experimental Neurophysiology, Fondazione Istituto Neurologico *Carlo Besta*, Milano, Italy; 2 Department of Neuroscience and Brain Technologies, Istituto Italiano di Tecnologia, Genova, Italy; 3 Department of Chemistry, Materials and Chemical Engineering *Giulio Natta*, Politecnico di Milano, Milano, Italy; 4 Scientific Direction Unit, Fondazione Istituto Neurologico *Carlo Besta*, Milano, Italy; 5 Department of Bioengineering, Politecnico di Milano, Milano, Italy; Aston University, United Kingdom

## Abstract

The passage of ions across biological membranes is regulated by passive and active mechanisms. Passive ion diffusion into organs depends on the ion-pairing properties of salts present in the serum. Potassium ions could affect brain activity by crossing the blood-brain barrier (BBB) and its accumulation in the extracellular cerebral space could precipitate seizures. In the present study, we analyze passive diffusion of a series of potassium salts in the *in vitro* isolated guinea pig brain preparation. Different potassium counter-anions confer ion-pairing and lipophilicity properties that modulate membrane diffusion of the salt. Extracellular recordings in different cortical areas demonstrated the presence of epileptiform activities that strongly relate to anion identity, following the qualitative order of the Hofmeister series. Indeed, highly lipophilic salts that easily cross the BBB enhanced extracellular potassium concentration measured by ion-selective electrodes and were the most effective pro-epileptic species. This study constitutes a novel contribution for the understanding of the potential epileptogenicity of potassium salts and, more generally, of the role of counter-anions in the passive passage of salts through biological membranes.

## Introduction

The passage of ions across biological membrane is regulated by active and passive mechanisms. In the central nervous systems, brain parenchyma is separated by the blood stream through the blood-brain barrier (BBB), formed by endothelial cells connected by tight junctions and resting on the basal lamina, pericytes and smooth muscle cells, astrocytes endfeet covering >98% of the vascular wall and occasional neuronal terminals [1-3]. BBB cells form a complex and fine-tuned transport machine that balances the influx of nutrients and the efflux of catabolites, toxins and drugs to maintain the Central Nervous System (CNS) homeostasis [4]. Endothelial BBB cells are highly polarized: transporters involved in the influx/efflux of various essential substrates such as electrolytes, nucleosides, amino acids, and glucose are distributed along the abluminal and luminal membranes. Transport mechanisms can be either carrier-mediated (facilitative) or ATP-dependent (active) and several physiological and pathological factors regulate BBB permeability by modulating membrane transporters, transcytotic vesicles and transcellular permeability [5,6]. 

Most ions diffuse passively across the BBB and their flow can be accelerated by partial association between anions and cations to form neutral ion-pair species in solution. Ion-pairing phenomena, first envisaged by Arrhenius at the end of 18^th^ century [7], are thoroughly studied especially to predict the tendency of specific anions and cations to associate in solutions. Little is known about ion-pairing in biological systems and about how ion-pairing influences passive ion transfer across biological membranes. In water, the propensity of ion-pairing is related to the balance between two counteracting effects: i) the ability of a given ion to favourably interact with water molecules and ii) its ability to interact with its counter-ion. Both energetic terms must confront with the intrinsic water/water interactions that must be overcome for effective solvation to occur. The propensity of ion-pairing can be expected to increase with the lipophilicity of the counter anion. This means that lipophilic anions (large and charge diffuse) present a degree of ion-pairing significantly higher than that of smaller halides or acetate salts. Ion-pairing attitude of different salts follows the anion Hofmeister series, a trend historically derived from the specific ability of different salts to precipitate egg-white proteins [8-12]. Hofmeister series simply orders ions as a function of their charge density, and consequently, of their water affinity. 

Understanding ion-pairing performance of salts in organic fluids, such as plasma, and across organic membranes, such as blood-organ partitions, could contribute to develop rational methods to deliver ionic compounds with therapeutic action more effectively and it could also help to understand side effects of exogenously applied compounds. The present report is the first attempt to study ion-pairing and transport phenomena of a series of potassium (K^+^) salts across the BBB. BBB integrity in the *in vitro* isolated guinea pig brain model utilized in the present study was confirmed by electron microscopy studies [13] and by functional analysis [14]. We focused on K^+^ , since its accumulation in cerebral extracellular space enhances neuronal excitability and may induce seizures by slowing down action potential repolarization [15,16]. When K^+^ reaches values of 5-6 mM, epileptic seizures may occur and can be measured with electrophysiological techniques [17-20]. Studying the potential epileptogenic effects of arterial perfusion of various K^+^ salts, represents a powerful model to investigate the passage across the BBB of ion-pairs with different lipophilicity. Moreover, this study could identify ideal K^+^-pairing conditions that safely prevent intracerebral K^+^ accumulation. 

## Methods

Brains of adult Hartley guinea pigs (150-200g; Charles River, Comerio, Italy) were isolated and maintained *in vitro* according to the standard procedure [13,21-24]. Briefly, animals were anesthetized with sodium thiopental (125mg/Kg i.p., Farmotal, Pharmacia, Italy) and were trans-cardially perfused with a cold (10°C), carboxygenated (95% O_2_, 5% CO_2_) saline solution (ACSF, composition: 126 mM NaCl, 3 mM KCl, 1.2 mM KH_2_PO_4_, 1.3 mM MgSO_4_, 2.4 mM CaCl_2_, 26 mM NaHCO_3_, 15 mM glucose, 2.1 mM HEPES and 3% dextran M.W. 70000, pH=7.1). Following decapitation, brains were isolated and were transferred into the recording chamber. A **polyethylene** cannula was inserted into the basilar artery and brain perfusion with the above solution (15°C, pH=7.3) at 7 ml/min was restored through the preserved resident arterial system via a peristaltic pump (Minipulse 3, Gilson, France). The temperature of the solution was slowly (0.5°C/min.) raised to 32°C to perform electrophysiological recordings. The study was carried out in accordance with the recommendations in the Guide for the Care and Use of Laboratory Animals of the National Institutes of Health. The protocol was approved by the Committee on Animal Care and Use and by Ethics Committee of the Fondazione Istituto Neurologico. Surgery and brain dissection were performed under sodium thiopental anaesthesia, and all efforts were made to minimize animal suffering.

Extracellular recordings were simultaneously performed in piriform cortex (PC), in medial entorhinal cortex (EC) and in the CA1 region of the hippocampus (hip) with micropipettes filled with 0.9 % NaCl (5-8 µm tip diameter, 5-10 MΩ resistance). During the experiments, field responses evoked by stimulation of the lateral olfactory tract (LOT) with a custom-made twisted silver wire were acquired via a multichannel Direct-Coupled (DC) amplifier (Biomedical Engineering, Thornwood, NY, US). 

Ion-sensitive microelectrodes (ISM) were utilized to record simultaneously ion-selective signal and field responses at the same cortical site (tip diameter 3±5 µm). The conventional electrode barrel was filled with 0.9% NaCl. The pipette barrel utilized for [K] measurements was filled at the tip with potassium iono phore I cocktail A (Fluka 60031) after 1 min exposure to dimethyldichlorosylane vapors (Fluka Germany) and was backfilled with 10 mM KCl following 2hr incubation at 120°C. [K]_o_ calibration solutions had the same composition of the solution used for arterial perfusion, except for KCl concentration, which was modified to obtain final K concentrations of 1, 2.5, 6, 12.5, and 48.2 mM. The absolute [K] values recorded during the experiment were obtained by solving the equation *y*= (*a* + *b*)*logx*, where *x* is the [K]_o_, *y* is the measured voltage reading induced by the changes in [K], and *a* + *b* is the slope coefficient derived from the calibration curve for each [K]_o_ sensitive electrode. Only microelectrodes with a response of 30 ±40 mV for 10 mM of [K] were utilized. Ion-selective and field DC signals were amplified with a high-input impedance head-stage amplifier (Biomedical Engineering, Thornwood, NY). Subtraction of the field potential from the K-sensitive electrode voltage reading was performed by the amplifier circuit.

Data were digitized with an AT-MIO-64E3 National A/D Board (National Instrument, Milan, Italy) and were analyzed with custom-made software (ELPHO^®^) developed in Labview by Vadym Gnatkovsky.

### Perfusion protocol

**Figure 1:**
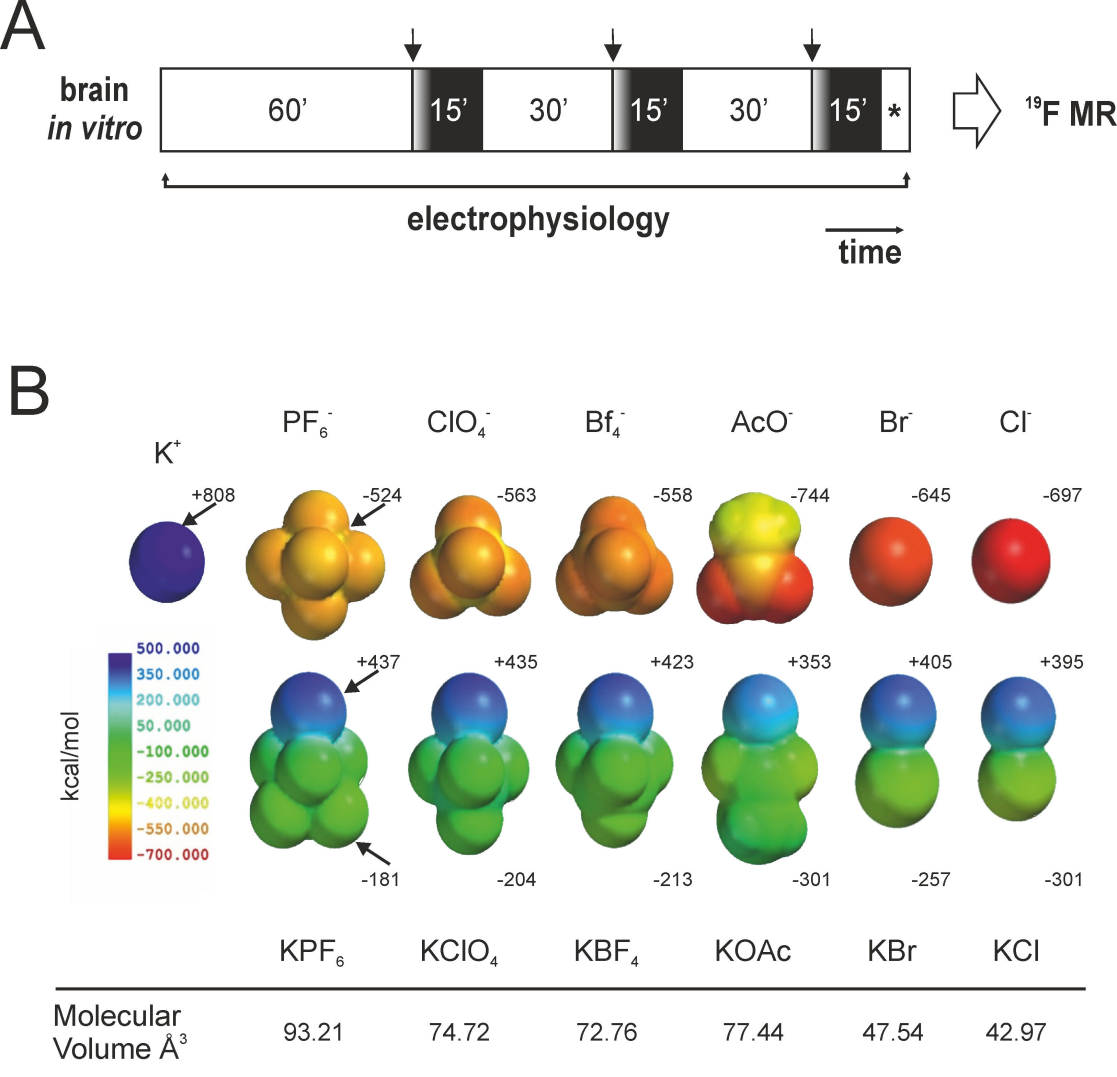
A. Schematic protocol of the experiments. Electrophysiological recordings are performed for the entire experimental period Each perfusion of salts lasted 15 min. and was followed by 30 min. wash-out. Brain tissue samples were than collected and processed for the ^19^F MR spectroscopy. **B:** Structures of KX salts (X= PF_6_^-^, BF_4_^-^, ClO_4_^-^, Br^-^, AcO^-^ and Cl^-^) and electrostatic potential surfaces (B3LYP/6-311+G**) of individuals ions and ion pairs. Blue regions indicate positive potentials and red regions negative ones. Maximum values of the electrostatic potential, at K atom, and minimum values, at O, F, Br and Cl atoms are indicated besides each structure. Molecular volumes are also reported (Å^3^).

The following K^+^ salts were selected for the study: KPF_6_, KClO_4_, KBr, KBF_4_, CH_3_CO_2_K (KOAc) and KCl. Each salt was diluted in the standard perfusion solution at different concentrations: 4.2, 8, 14.2 mM (KPF_6_, KClO_4_, KBr, KBF_4_, KOAc and KCl) and 20 mM (KBr, KBF_4_, KOAc and KCl). Each perfusion lasted 15 min and was followed by a 30 min wash-out (Figure 1A). Successive perfusions in the same experiment were repeated using descending concentrations of the same K^+^ salt. Since the main goal of the study was to establish the effects of different K^+^ salts on cellular excitability, KCl was completely removed from the standard solution and KH_2_PO_4_ was substituted with NaH_2_PO_4_. Osmolarity was maintained by isotonic reduction of NaCl to 114.8 mM and 109 mM, respectively, when 14.2 and 20 mM K^+^ solutions were used. 

### Stimulation protocol

To evaluate cortical excitability before, during and after K^+^ salts perfusion, we performed pair pulse (PP) LOT-stimulation protocols with inter-stimulus intervals of 40 and 100 msec, separated by 5 sec. PP tests were delivered every 20 sec during perfusion of each salt, and every 60 sec during the rest of the experiment. We compared amplitude and morphology of conditioned responses evoked by LOT stimulations before, during and after K^+^ salts perfusion. The values are reported as means ± standard error of mean (SEM), if not stated differently. 

### Molecular electrostatic potential (MEP) study

Geometry optimizations of the KX salts (X= PF_6_^-^, BF_4_^-^, ClO_4_^-^, Br^-^, AcO^-^ and Cl^-^) structures were carried out in vacuum using Density Functional Theory (DFT-B3LYP functional) and the 6-311+G** basis set for all atoms. Maps of the MEP were plotted onto the van der Waals surface for each species and shown in Figure 1B. Molecular volumes were calculated in Å^3^. The different values of the electrostatic potential at the surface are represented by different colours and expressed in kcal/mol; red represents regions of most negative electrostatic potential, blue represents regions of most positive electrostatic potential (Figure 1B). Calculations were computed with the Spartan ’08 software package. 

### ^19^F Magnetic Resonance (MR) spectroscopy

To verify diffusion of K^+^ salts across the BBB in brains perfused with KPF_6_ (14.2 mM) and KBF_4_ (20 mM), samples from hippocampus and EC of defined volumes (V_B_) were collected after 2 mins of wash out at the end of the electrophysiological experiment in order to exclude the salts from the vessels. Then, they were introduced into the MR tube by a syringe whereupon a known volume of D_2_O (V_W,_ between 250 and 500µL) was added to each sample. Tubes were sonicated for 10-20 minutes, and after 1 hour, the final volume of the sample (V_F_) was calculated by measuring the height of the meniscus on the top of the water layer in the tube. Tissue volume was then calculated as V_B_ = V_F_-V_W_. 10µL of a solution containing CF_3_COONa (0.6 M) were then added to the sample as internal reference. ^19^F-NMR spectra were recorded on a Bruker Advance 500 MHz (SW = 200 ppm, D1 = 5 s; LB = 1, T = 300 K, NS = 32 or 64). The concentration of PF_6_^-^ and BF4^-^ were obtained by integral analysis of the analytes peaks compared to that of the reference (CF_3_- group).

## Results

We previously demonstrated that BBB in the *in vitro* guinea pig brain is structurally and functionally preserved [14,25]. We tested BBB permeability to a series of potassium salts (KPF_6_^-^, KClO_4_, KBr, KBF_4_, CH_3_CO_2_K (KOAc) and KCl; Figure 1B) having different lipophilicity by evaluating their effects on network excitability. A given salt species can be considered in equilibrium between its solvated/separated and ion-paired states. Passage through membrane can ideally occur in both states. However, from simple considerations on the effective charge, a higher permeability through a lipophilic membrane is expected for neutral species than for individual ions. As for ion-pairing equilibrium, both thermodynamic and kinetic aspects of the in/out process crucially depend on ions identity. Therefore, passive passage through the BBB membrane is expected to be higher for K^+^ salts with a lipophilic counter-ion. To further clarify which ion-pair would be more prone, from a theoretical point of view, to diffuse through the BBB, molecular electrostatic potentials (MEP) were calculated by DFT method. MEP calculation (Figure 1B, bottom part) informs about charge density distribution of salts and on the effect of ion pairing. As expected, the ion-pair formation lowers the absolute values of the electrostatic potential for both the cation and the anion, i.e., the ion-pair is less polar than individual ions (Figure 1B).

**Figure 2:**
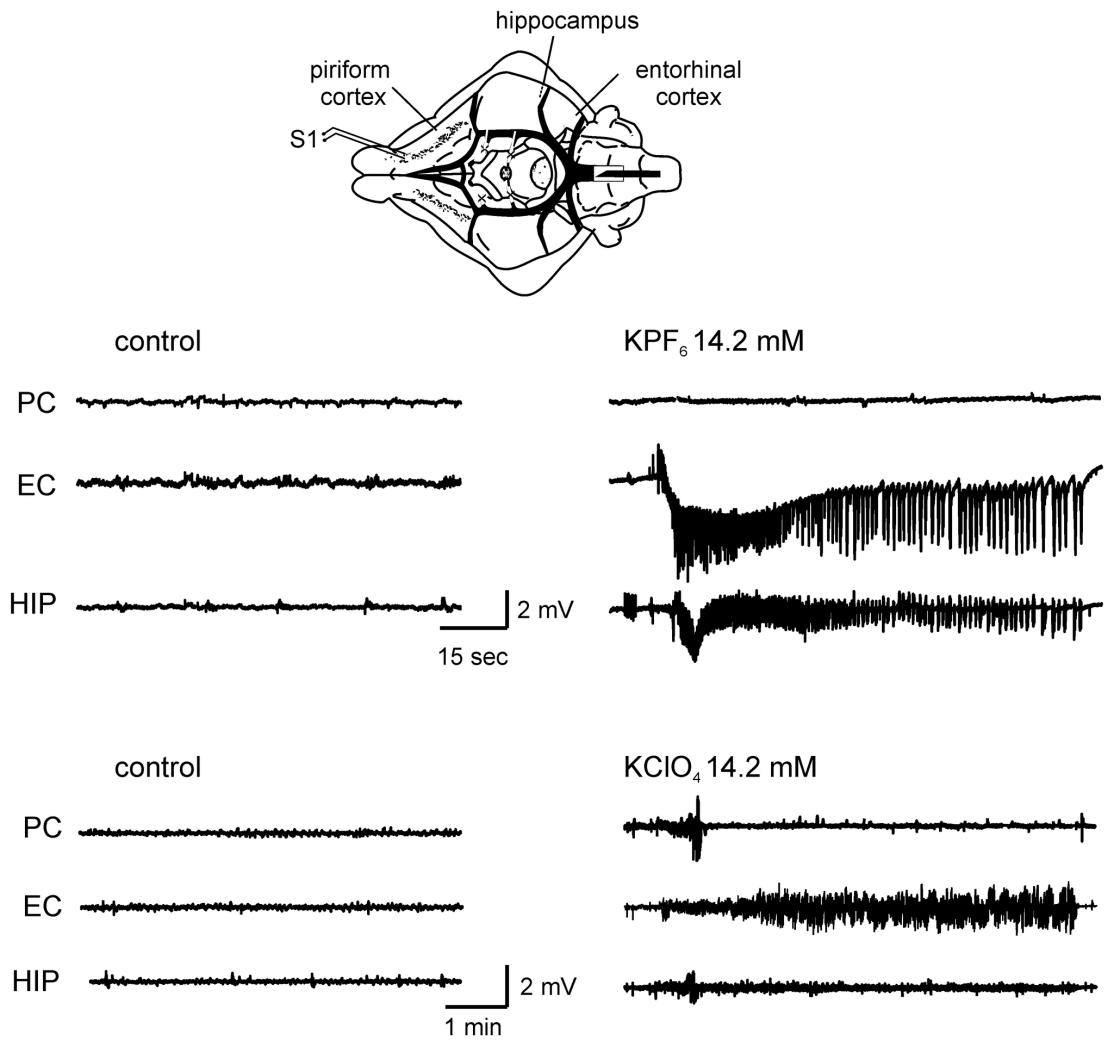
Effects of perfusion of K^+^ salts in the *in* *vitro* isolated guinea pig brain. A scheme of the position of the recording and stimulating (S1, on the lateral olfactory tract) electrodes is shown in the upper part of the figure. The upper set of traces illustrates the effect of 14.2 mM KPF_6_ perfusion (right) compared to control (left); seizure activity was induced by this salt. In the lower part of the figure the effect of perfusion with 14.2 mM KClO_4_ is shown. PC: piriform cortex; EC: entorhinal cortex; HIP: CA1 region of the hippocampus.

**Figure 3:**
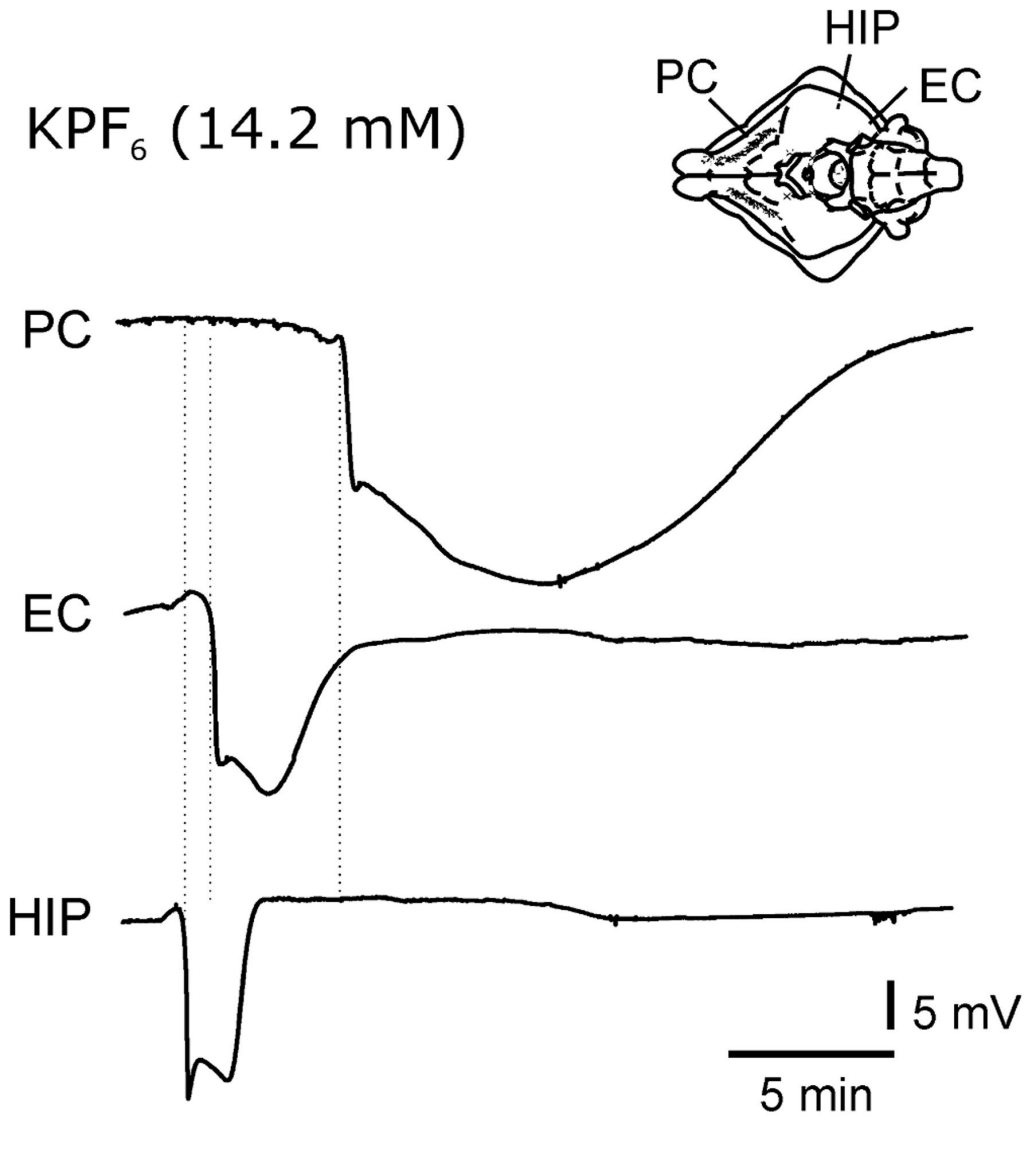
KPF_6_ 14.2 mM perfusion induced large amplitude depolarization events that propagated through the cortical mantel, from the hippocampus to the entorhinal (EC) and piriform cortex (PC). These events were interpreted as spreading depolarization phenomena (Somjen 2001).

**Figure 4:**
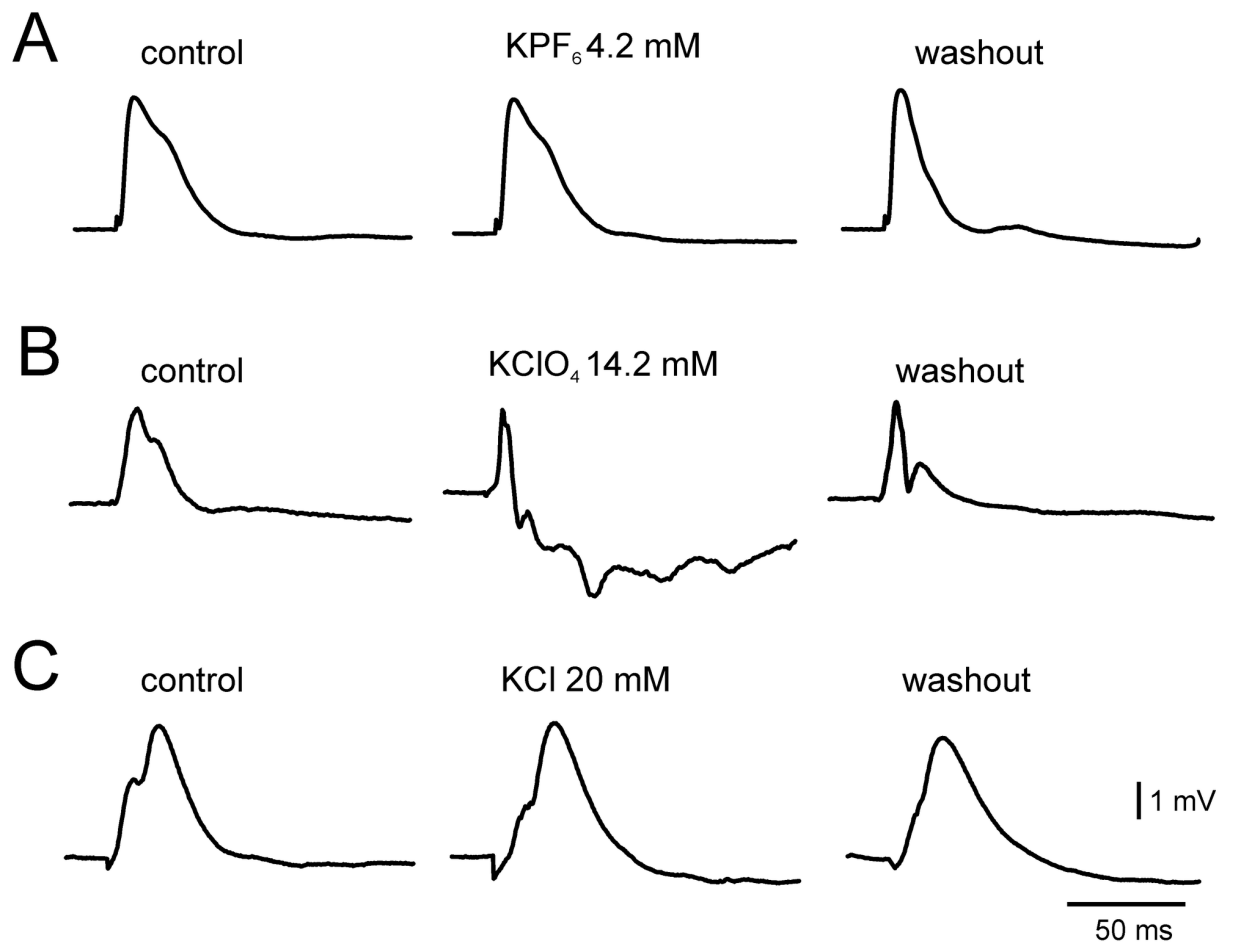
Effects of 4.2 mM KPF_6_ (A), 14.2 mM KClO_4_ (B) and 20 mM KCl (C) on the field potentials evoked in the piriform cortex by lateral olfactory tract (LOT) stimulation. Left, middle and right traces: before, during and after washout of the K^+^ salts, respectively.

The most lipophilic salt, KPF_6_, at concentration of 8 mM to 14.2 mM always induced one or more seizure-like events (1.84, ±1.056 n=10) recorded in all recorded structures (Figure 2). Seizures started in EC 3.98±1.94 (mean±SEM) minutes after KPF_6_ perfusion onset and propagated to both hippocampal formation and PC. In EC these events lasted 2.91 ± 2.22 min. In 40% of the experiments, after the ictal event spreading depolarization (SD, [26]) characterized by an abrupt voltage drop in the extracellular potential (11.8 mV, ± 1.8; n= 4) were also recorded (Figure 3). SDs appeared 4 mins after the end of the seizure and lasted 3 mins (± 0.9). At lower concentration (4.2 mM), KPF_6_ neither induced seizures and nor altered evoked activity (Figure 4A).

**Figure 5:**
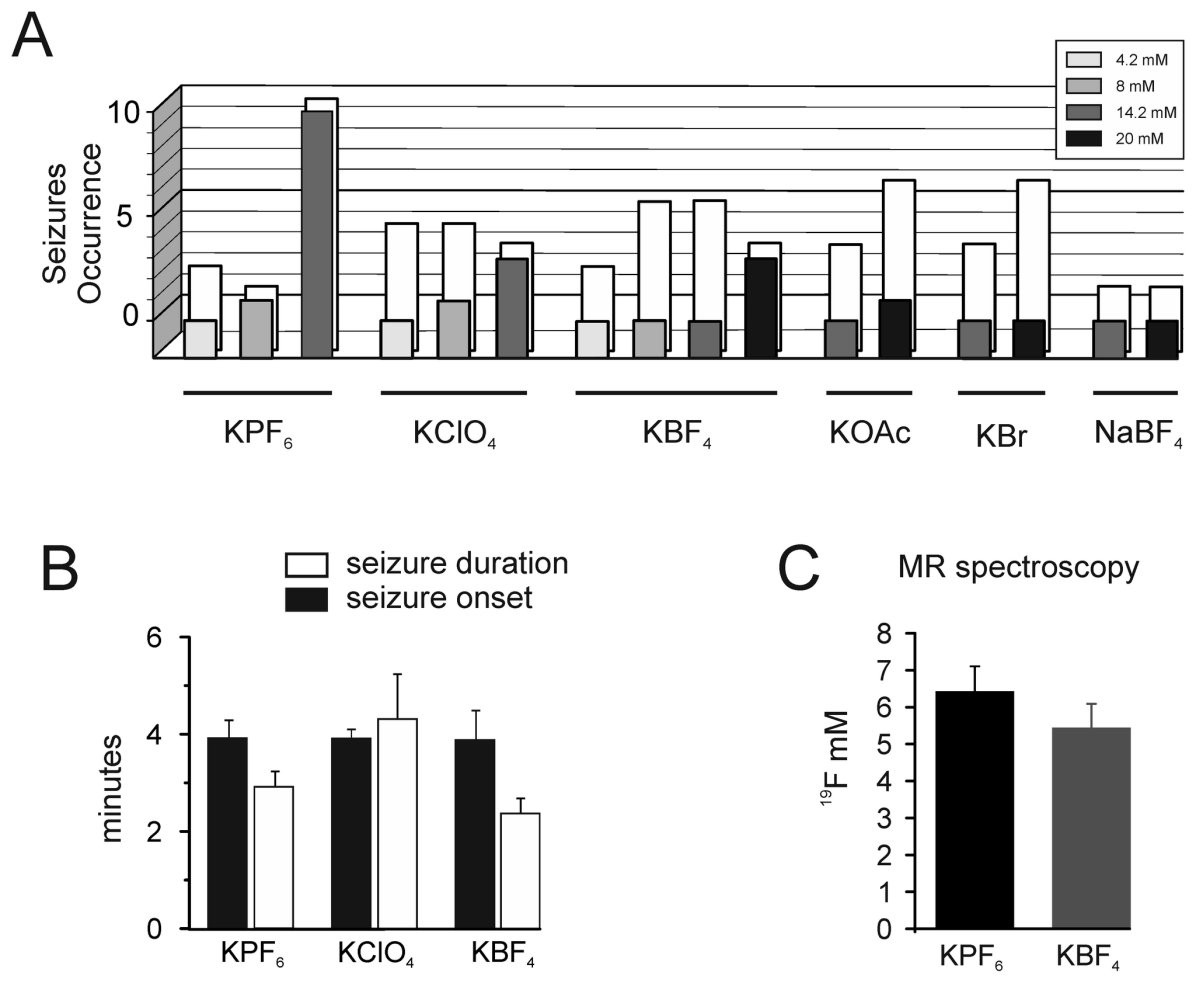
A: Seizure occurrence after perfusion of the *in* *vitro* isolated guinea pig brain with different K^+^ salt solutions. The white columns represent the total number of experiments. The light grey, dark grey and black columns mark the effects of the perfusion of K^+^ salts at 8, 14.2m 20 mM, respectively. **B**: Time at onset (black columns) and duration (white columns) of seizure activity after perfusion with 14.2 KPF_6_, 14.2 mM KClO_4_ and 20 mM KBF_4_. **C**: Brain parenchyma concentration of two salts (KPF_6_ and KBF_4_) estimated by ^19^F MR spectroscopy on CA1-EC specimens, collected after arterial perfusion with 14.2 mM KPF_6_ (n= 4; black column) and 20 mM KBF_4_ (n= 3; grey column).

By changing the counter-anion identity, we noticed that the induction of seizures strictly correlated with high lipophilicity of the salt. Considering the following Hofmeister series for anions: PF_6_^-^, ClO_4_^-^, BF_4_^-^ and CH_3_CO_2_^-^, Br^-^ and Cl^-^, a more marked lipophilicity of the counter-anion resulted in a more evident enhanced excitability, while no changes occurred with more hydrophilic salts. Indeed, KBF_4_ and KOAc provoked ictal events less frequently than KPF_6_ (67% and 16.7% of experiments, respectively) and only when their concentration was increased to 20 mM (Figure 5A). The time at onset and the duration of seizure-like activity induced by 14.2 and 20 mM K^+^ salts were not different (Figure 5B). Interestingly, the perfusion of the brain with the same concentrations of a salt, in which Na^+^ was substituted for K^+^ (for example, NaBF_4_), did not induce seizure activity (Figure 5A). KBr, which stands on the hydrophobic side of the series, never induced changes measured by electrophysiological means, even at 20 mM concentration (Figure 5A). 

In the attempt to elucidate the diffusion through the BBB of K^+^, apparently mediated by an ion-pairing related mechanism, we perfused the *in vitro* isolated brain with a solution containing KCl 20 mM. We used this concentration based on previous findings that in a structurally and functionally preserved BBB the potassium membrane transport dampened the inflow of K^+^ during high KCl perfusion [14]. During KCl perfusion we never recorded seizure-like events or hyperexcitable evoked responses comparable to that induced by 14.2 mM KClO_4_ (Figure 4C). However, in structures more prone to develop synchrony, such as the hippocampus, KCl induced bursting-like activity (not shown). The average duration of this pattern was of 4.85 ± 4.61 min (n=3). Changes in extracellular potassium concentration were measured with ISMs bilaterally positioned in ECs during the perfusion of two potassium salts with opposite positions within the Hofmeister serie, KClO_4_ (14.2 mM; n= 3) and KCl (14.2 mM; n=6). The perfusion of KClO_4_ induced an increase in extracellular potassium from 4.2 mM to 8.91 mM (±0.21) of [K]_o_ after 5.6 min ( ±1.3). On the top of it, ictal (n=4) or spreading depression (n=2) -like events were observed. Seizures and spreading depressions further increased [K]_o_ by 5.05 mM (± 0.04) and 22.36 mM (±3.73), respectively. Unlike KClO_4_, KCl perfusion only slightly increased [K]_o_ to 4.8±0.5 mM (mean±SD) and neither seizure-like nor spreading depression-like events were observed. These data demonstrate that ionic K crosses the BBB and accumulates in the brain parenchyma when it is coupled to a weak ion pair. 

In the case of PF_6_^-^ and BF_4_^-^ salts, we further verified the actual transport in the brain parenchyma across the BBB by performing ^19^F MRI spectroscopy measurements on brain samples. The analysis of specimens collected from different brain regions (hippocampus and entorhinal cortex) revealed the presence of fluorinated species (PF_6_^-^ and BF_4_^-^) in the brain with a mean concentration of 6.42 mM (n= 9; ±0.68) and 5.44 mM (n= 6, ±0.65), respectively (Figure 5C).

## Discussion

In the present study, we analyze passive diffusions of a series of K^+^ salts perfused by arterial route in the *in vitro* isolated guinea pig brain preparation and evaluated K^+^-induced changes in excitability as parameter of diffusion efficacy. We show that the phenomenon of ion-pairing modulates the transport of charged ion species through the BBB and regulates the access of ionic xenobiotics to the brain parenchyma. Ion pairing represents the formation of a neutral adduct made of a pair of oppositely charged ions held together by Coulomb attraction. The extent of ion-pairing of a given salt is dependent on many factors, such as solvent, ionic strength and above all on the ions’ identities. Once formed, the ion pair is more lipophilic than the separated ions and this can have a role in the passage of salts through biological lipophilic membranes, such as BBB. More specifically, ion pairing can improve the permeability of ionic paired moieties across the BBB. It is interesting to note that the observed trend in ion-pair transport through the BBB derived from the electrophysiological study (KPF_6_ > KClO_4_ > KBF_4_ > AcOK, KBr > KCl) follows that observed for ion-pair MEP values calculated by DFT method. AcOK is an exception to this trend, having a higher maximum negative potential than KBr, but showing a slightly more pronounced transport through the BBB. This particular case indicates that the use of a single physical parameter to describe a complex phenomenon has limitations. This notwithstanding, the behaviour of AcOK can be easily explained as a consequence of the less symmetric distribution of charge and of the marked difference in molecular volume between AcOK and KBr. The acetate salt is almost 50% bigger than KBr and, therefore, it possesses an overall lower charge density. This may represent the rationale behind its higher diffusion through the BBB.

We exploited the effect of extracellular K^+^ on brain excitability [27,28] to test BBB permeability of different ion-paired salts. We assumed that a high permeability of the K^+^ salt induced stronger enhancement of brain excitability, expressed by epileptiform discharges. Perfusion of solutions containing ion pairs composed of more lipophilic ions, which more effectively pass the BBB, increase the concentration of K^+^ inside the brain (as demonstrated by ISM experiments) and induce hyperexcitability changes evaluated by LOT stimulation that may lead to epileptiform discharges. We hypothesize that the anion transfer and the stochastic fission of the pair induce a remarkable increase of K^+^ in the brain extracellular space. These reliable and reversible effects (i.e. seizures and dysinhibition of evoked responses), as demonstrated by the means of ISMs, were prominent for salts in the left of the Hofmeister series with a highly separated ionic state, that is responsible for an elevation of K^+^ in the extracellular space. KPF_6_ and KClO_4_ have more lipophilic counter-anions, easily cross the BBB and, as a result, they are the most effective in inducing seizures. The lack of both excitability changes and [K]_o_ increase during the arterial perfusion of elevate concentrations of KBr and KCl, points to a less effective delivery of the ion pair through the BBB. 

The specificity of K^+^ in the induction of hyperexcitability is confirmed by the demonstration that by substituting Na^+^ for K^+^ in the solution, for instance by perfusing NaBF_4_ as equivalent to KBF_4_, did not enhance brain excitability even when it was perfused at 20 mM concentration. Only in the case for NaPF_6_ hyperexcitability was observed, and we speculate that this could be due to a specific intrinsic effect of PF_6_^-^ anion on brain excitability (see below). 

In case of two fluoride salts, KPF_6_ and KBF_4_, it was possible to demonstrate accumulation of the salts into the brain parenchyma by performing ^19^F-MR spectroscopy of both intact brains and of homogenized brain tissue after perfusion of the two fluoride salts. The ^19^F isotope is a useful marker to study BBB permeability with MRI techniques in physiological conditions and during neurodegenerative processes [29-31], since there is no endogenous fluoride ion in brain tissue and thus the background signal from that tissue is virtually absent. Large quantity of fluoride tracer in the millimolar range are required for detection with MR. These potentially toxic concentrations [32] could be reached in our experiments because the isolated brain preparation is maintained *in vitro* and peripheral effects can be neglected. In different whole-cell patch clamp experiments, cesium fluoride was found to interfere with voltage-activated Ca^2+^ channels kinetics [33,34]. Moreover, fluoride complexes inhibit GTPases and ATPases, including Na,K ATPase [35-37], and could result in neuronal hyperexcitability. PF_6_^-^ inhibition of ouabain-sensitive Na,K ATPase located on endothelial cells interfere with the efflux of K^+^ from the brain to the lumen and may promote its accumulation in the extracellular cerebral space. In line with this hypothesis, perfusion of high concentrations of NaPF_6_ (14.2 mM) induced seizure activity (data not shown). The latter mechanism may reinforce K^+^ increase mediated by the anion-transfer, and hence force extracellular K^+^ above 10-15 mM, a concentration that may precipitate spreading depolarizations phenomena [26] as observed in our experiments. The dependence of the effects (i.e., increased excitation) on the concentration of (PF_6_^-^) explains why arterial perfusion with 4.2 mM of KPF_6_ did not modify brain excitability.

These results provide understanding of the mechanisms of ion-pair mediated membrane transport across the BBB. Moreover, different works have shown that BBB disruption causes seizures [38] and, to the other hand, its preservation prevents seizure development [39]. In this experimental model the functional consequences of the ion-paired membrane transport, when observable, were reversible and never deteriorated in a vasogenic edema. Thus this work emphasizes the potential of ion-pair approach to increase passive permeability of drugs [40,41] or other compounds to be delivered to the brain.
